# Quantifying and understanding the roles of diabetes educators in Malaysian primary health clinics: a mixed-methods study

**DOI:** 10.1186/s12913-026-14144-7

**Published:** 2026-02-03

**Authors:** Nur Jihan Noris, Pangie Bakit, Intan Syafinaz Saimy, Zalina Libasin, Ili Liyana Khairul Anuar, Wan Nur Adila Wan Alias, Sivarajan Ramasamy

**Affiliations:** 1 Institute for Health Management, National Institutes of Health Malaysia, Shah Alam, Malaysia; 2 Faculty of Medicine, Universiti Teknologi Mara, Sungai Buloh, Malaysia; 3 Non-Communicable Disease Unit, Negeri Sembilan State Health Department, Seremban, Malaysia

**Keywords:** Diabetes, Diabetes educator, Health clinic, Diabetes education

## Abstract

**Introduction:**

Diabetes educators (DEs) play a critical role in supporting self-management and improving clinical outcomes among people with diabetes. However, their roles within primary healthcare settings in Malaysia remain underexplored, particularly with respect to time allocation for diabetes care and the challenges they face.

**Methods:**

A mixed-methods cross-sectional study was conducted in two phases from January 2022 until August 2023. Phase 1 involved a time‒motion study of 51 DEs across 43 public primary care clinics in Negeri Sembilan, which were observed over five working days to quantify the time spent across different activity domains. Phase 2 consisted of three in-depth interviews and five focus group discussions with 27 purposively sampled DEs. The quantitative data were analysed via descriptive statistics, whereas thematic analysis was applied to the qualitative transcripts.

**Results:**

Five domains of DE activities were identified: diabetes care, non-diabetes care, administrative, supportive, and personal. DEs spent most of their time on diabetes care (63.3%), but two-thirds of this was indirect care, especially documentation. Non-diabetes responsibilities consumed approximately 26.5% of their time. The qualitative findings revealed three overarching themes influencing DE roles: organisational, patient-related, and personal factors. A central theme of resilience emerged, reflecting DEs’ coping strategies amid multiple roles demands, documentation burdens, and systemic constraints.

**Conclusions:**

This study provides new insights on how DEs allocate their time and challenges to Malaysia’s primary health clinics. Although they were able to dedicate substantial time to diabetes care, their effectiveness appeared to be hindered by administrative burden and role conflict. Targeted interventions are needed to streamline workflows, reduce documentation burdens by integrating information system, enhance leaders’ engagement in diabetes care, and clarify DE roles to strengthen diabetes care services in PHCs.

**Supplementary Information:**

The online version contains supplementary material available at 10.1186/s12913-026-14144-7.

## Background

Diabetes mellitus (DM) is a chronic metabolic disease characterized by elevated blood glucose levels and, over time, is associated with micro- and macrovascular complications. It has emerged as a pressing public health challenge globally, with a significant effect on patients’ quality of life. According to the World Health Organization (WHO), the number of people living with diabetes has steadily increased over the past few decades, with the number increasing from 200 million in 1990 to 830 million in 2022 [1], and diabetes was also the direct cause of death for 1.6 million patients in 2021 [1]. Globally, diabetes incidence has quadrupled since 1990, affecting more than 800 million people and causing more than 1.6 million deaths annually. The burden falls disproportionately on low- and middle-income countries (LMICs), where more than half of people with diabetes remain untreated [1]. In addition to individual health, diabetes imposes significant economic strains on families, communities, and healthcare systems worldwide [2, 3].

Efforts to mitigate this burden emphasize early detection, comprehensive treatment, and the critical role of education in empowering patients to manage their conditions effectively [4]. With a healthy diet, regular physical activity, medication, and regular screening and treatment for complications, the consequences of diabetes can be avoided or delayed. While advancements in pharmacotherapy have improved glycemic control outcomes, there remains a substantial gap in achieving optimal diabetes management owing to behavioral, social, and systemic barriers. Structured interventional programmes designed to promote self-management and improve clinical outcomes, such as the Diabetes Self-Management Education and Support (DSMES) programme in the United States [5, 6] and the Diabetes Education and Self-Management for Ongoing and Newly Diagnosed (DESMOND) programme in the United Kingdom [7, 8], have demonstrated measurable success in equipping individuals with the knowledge and skills necessary for effective diabetes management. Recent systematic reviews and meta-analyses have demonstrated that structured education interventions, particularly those led by trained educators or nurses, reduces glycated hemoglobin (HbA1c) by approximately 0.5% to 1.0**%** [6, 9, 10], compared to standard care. Beyond clinical markers, these interventions are associated with improved health-related quality of life (HRQoL), enhanced self-efficacy, and a reduction in diabetes-related distress [11]. The effectiveness of these programs is often tied to the professionalization of the educator role. In health systems where diabetes education is well-established, such as Australia, DEs are recognized as Credentialled Diabetes Educators (CDEs) which is a formal title requiring post-graduate qualification and clinical expertise [12]. Similarly, in the United Kingdom, the role is integrated within a national quality assurance framework to ensure standardized care delivery [13]. These international models highlight a clearly defined role and professional pathway that helped improve clinical outcomes. However, the implementation of such structured roles in many other regions, particularly LMICs including Malaysia remains constrained by limited resources and a lack of formal recognition [14, 15].

In Malaysia, diabetes management has shifted from treatment-centered to patient-centered approach emphasizing education and self-care [16]. Diabetes management is predominantly delivered through public primary healthcare clinics (PHCs) [4]. In response to the growing burden of diabetes [4], the Ministry of Health (MOH) introduced several initiatives, including the National Strategic Plan for Non-Communicable Diseases (NCD) [17]and the Enhanced Primary Healthcare (EnPHC) programme, which emphasise multidisciplinary, team-based management of chronic diseases within primary care [18, 19]. Within these frameworks, diabetes education and self-management support constitute core components of diabetes care, and diabetes educators contribute to patient education, follow-up, and elements of care coordination as part of broader primary healthcare teams. This practice is supported by the Malaysian Diabetes Education Manual (2020) [20] and the Clinical Practice Guidelines for Type 2 Diabetes Mellitus (2020) [16], which outline the scope of diabetes education and emphasise the delivery of structured education. However, recent evaluations of the EnPHC have shown that while process-of-care indicators improved, achieving optimal clinical outcomes remains challenging due to resource and staffing constraints [21].

DE role in Malaysia began in 2004 with the introduction of a six-month specialized training program by MOH for nurses and Assistant Medical Officers (AMOs). This was later evolved into a one-year Advanced Diploma in Diabetes Care in 2019. As of 2022, from unpublished data from Non-Communicable Disease Division, MOH, there were 1,304 trained DEs nationwide, with over 62% stationed in primary care to manage the high patient burden. Their responsibilities include conducting individual and group education sessions, facilitating insulin initiation and titration, and providing ongoing support to help patients overcome barriers to effective self-management [20, 22]. The potential impact of these roles is significant, as recent local data from a single-centre Nurse Educator-Led Clinic (NELC) in Selangor demonstrates that focused educational interventions can reduce mean HbA1c from 9.80% to 7.68% over six months [23]. Despite their critical role, DE in Malaysia lacks formal recognition by a national certification body, distinguishing it from international models such as Australia and United Kingdom. Furthermore, local studies highlighted DEs in Malaysia facing unique challenges including high patient-to-DE ratios, limited time for education delivery, a lack of clear guidelines, the absence of audits or surveillance, variations in clinic-specific delivery systems and resource constraints [15]. The lack of clear and standardized guidelines contributes to variations in the quality and consistency of care [4, 22]. While human resource constraints led them to prioritise general clinical duties over their intended roles and responsibilities as a DE [15].

Despite the acknowledged importance of DEs, no specific study in Malaysia has quantitatively assessed their time distribution or explored the factors affecting their capacity to perform diabetes care duties. This study therefore aimed to quantify the time DEs spent on diabetes-related and non-diabetes-related tasks, and to explore barriers and facilitators to their role in primary healthcare. The quantitative findings revealed portion of DEs’ time devoted to diabetes and non-diabetes activities. These results informed the subsequent qualitative phase, which explored the underlying reasons and challenges influencing DEs’ role performance. This study was conducted in Negeri Sembilan—a state with one of the highest diabetes prevalence rates in Malaysia [24](. In this state, challenges for DEs are particularly pronounced due to the high diabetes prevalence, necessitating the need for tailored strategies to optimize the utilization of DEs and support their professional development. By measuring the time spent and exploring factors affecting their roles, this research aimed to provide valuable insights into the challenges and opportunities for optimizing diabetes education in this high-prevalence region. Focusing on a region with a high burden of diabetes allowed this research to inform policy decisions and support the development of sustainable and actionable strategies for improving diabetes care delivery, both in Negeri Sembilan and Malaysia as a whole.

### Study design and setting

This was a mixed-methods study with an embedded design integrating both quantitative and qualitative approaches conducted in two sequential phases. The rationale for doing the mixed qualitative and qualitative study is that neither quantitative nor qualitative are sufficient by themselves to capture the gravity and details of the situation. The quantitative observations provided measurable information on how DE spent their time and performed their tasks, while the qualitative interviews helped explain and give context to these findings by exploring their experiences and views in more depth. Combining both approaches allowed for a more complete understanding of their roles and challenges in primary healthcare. In phase 1, the quantitative approach measured the time utilization by 51 DEs in 43 primary clinics. It focused on diabetes care aspects while also measuring other activities performed in the clinic that were unrelated to diabetes care. Phase 2 was conducted to build upon the mean and proportion of time spent on diabetes care, analysed from Phase 1. This second phase aimed to explore the underlying factors that contributed to these patterns of time use, focusing on the facilitators and barriers influencing DEs’ daily practice and role definition within PHCs. Qualitative data were collected through focus group discussions (FGDs) and in-depth interviews (IDIs) to explore the factors influencing DEs’ ability to allocate time for diabetes care. FGDs were conducted with DEs grouped by years of experience and profession, while IDIs involved senior DEs in managerial roles. Segregating participants in this way was intended to encourage more open expressions of views from junior DEs. The interviews were conducted in both the Malay and English languages. The study was registered under the National Medical Research Register, Ministry of Health Malaysia (NMRR-21-1045-59255) and was approved by the Medical Research and Ethics Committee (MREC) MOH Malaysia.

### Study sampling

For phase 1 (quantitative phase), universal sampling was applied, where all DEs in PHCs who provided consent for observation were recruited. A total of 51 out of 56 DEs participated, representing a 91.1% response rate. The sample included DEs from clinic Type I, II, III, IV, V and VI to ensure representation across service settings. As depicted in Table 1, most participants were from Type III clinics, due to the higher number of Type III clinics in the state. These clinics were categorised based on their daily patient attendance, using the most recent list obtained from the Negeri Sembilan State Health Department. Although clinics varied in size and patient volume, the objective was not to compare time utilization between clinic types but to capture a comprehensive picture of DE activities across diverse clinic settings. In phase 2 (qualitative phase), the DEs were purposively sampled to ensure that the maximum variation was covered. The inclusion criteria were as follows: (i) profession; (ii) years of service such as DE, divided into senior > 8 years and junior < 2 years; and (iii) their time spent in diabetes care, i.e., those with the least time spent, the median and the greatest amount of time spent.

### Data collection methods

During Phase 1, a time‒motion study employing continuous direct observation methods was conducted. Each DE was shadowed by the same trained observer for five working days, from the start until the end of each working day. The observers had undergone data collection training prior to the actual data collection. During the five days, the observer captured each task’s start and end time and described the tasks performed in a predefined observation sheet, which were later entered into MS Excel. The pre-designed observation sheet was developed by the investigators and was not adapted from any existing tool, nor has it been published or used by other prior study. The observation sheet was piloted at three non-sample clinics to ensure consistency of data collection. During the pilot, each observer was paired with an investigator to observe the same DE, and the findings were cross-checked and discussed to standardize the observation approach, interpretations and clarify any discrepancies. As most of the data collectors were more comfortable and fluent in Malay language, the tool was developed mainly in Malay language. To expedite the data collection, the earlier phase of this study developed a preliminary taxonomy enlisting the major domains of activities in the clinic and their subdomains. This taxonomy was modified and further enriched after observations of all 51 DEs were completed. The observations were performed in the natural work setting of the participants, and the observers did not interfere with the activities performed by DEs or their patient interactions. Clarifications were made after the tasks were finished and the patient left the room. The DEs were not observed during their one-hour break, as it was not relevant to the study and represented a personal break for them. The observation data in MS Excel were emailed daily to the investigators and validated by the principal investigator (PI).

In Phase 2, after a preliminary analysis of the time spent by each DE for diabetes care and other non-diabetes tasks, five focus group discussions (FGDs) and three in-depth interviews (IDIs) were conducted with 27 DEs. Participants were purposively sampled and categorized into homogenous groups of four to five members. Categorization was based on three criterias: profession, years of experience as DE, and proportion of time spent for diabetes care, as analysed from Phase 1.

First, groups were first divided between profession of either Nurses or Assistant Medical Officers. Then, regarding years of experience, participants were separated into three groups: those with < two years of experience, three to seven years, and > eight years. Finally, participants were grouped by the proportion of time dedicated to diabetes care: < 50%, 60%–80%, and > 80%. The specific criteria for group division were not disclosed to participants prior to the sessions. This was done to encourage natural, spontaneous interaction and to ensure that participants’ contributions were not influenced by their awareness of the predefined categories.

**Fig. 1:**
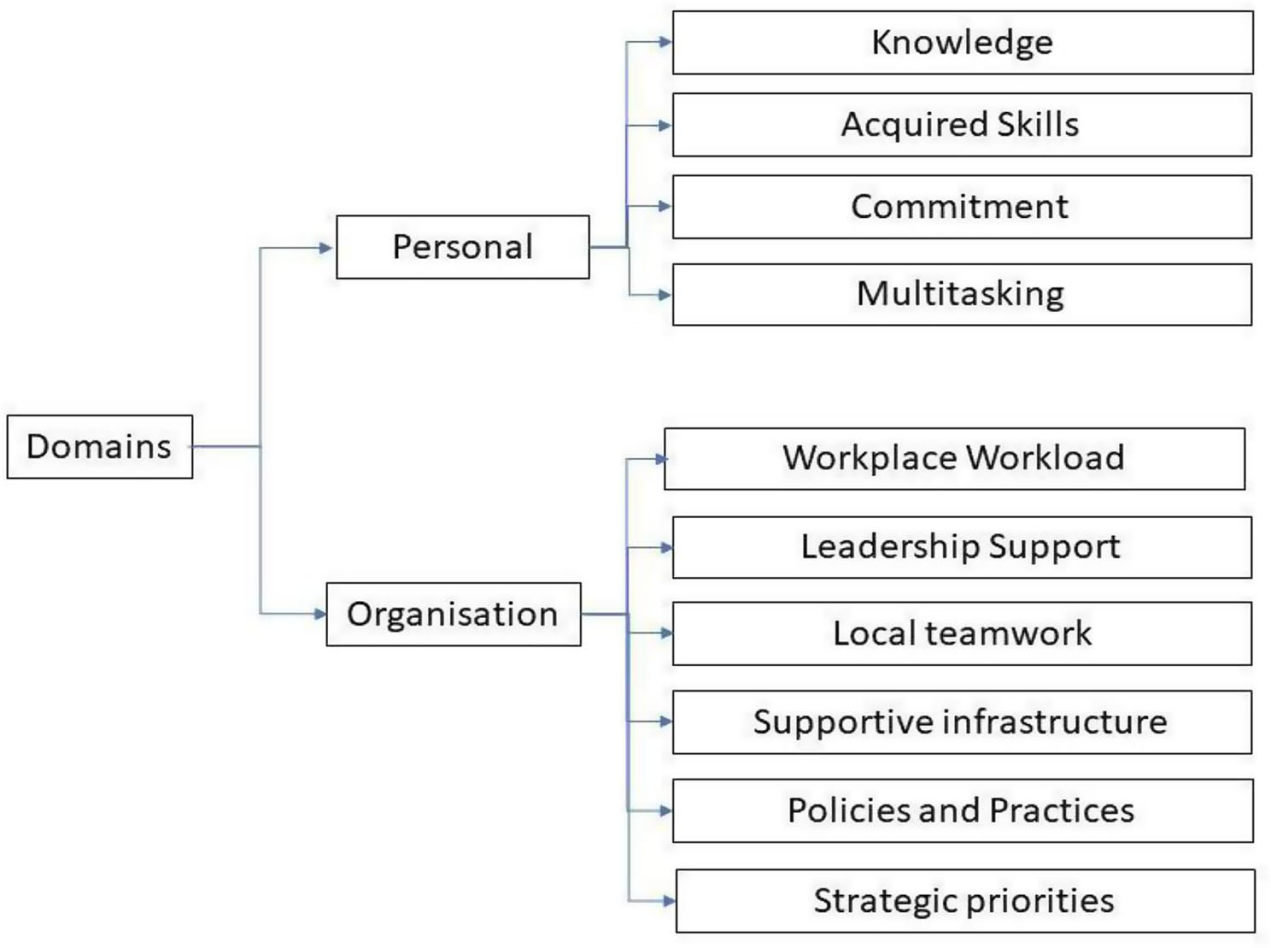
Conceptual framework on factors affecting time spent by DE

The interviews were assisted by a semi structured guide (Appendix A). The guide was developed by adapting it to the framework used in this study (Fig. 1). This guide has not been used or published elsewhere in prior study. Each FGD lasted 60 to 90 min, whereas IDIs took approximately 45 to 60 min. The FGDs and IDIs were conducted in meeting rooms in their state health office, away from their workplace, to minimize interruptions. The interviews were conducted in Malay and English to facilitate expression and were moderated by two researchers with no prior relationship with the participants. Confidentiality was assured beforehand, with participants signing a consent form and confidentiality reaccentuated during the interview sessions. All sessions were audio-recorded for transcribing and analysis purposes. In each session, participants were prompted about their experiences, challenges and facilitators in their daily task of providing diabetes care.

### Data management and analysis

The observation data were first recorded in a data collection book. These data were then tabulated into Microsoft Excel version 2021 for data cleaning and validation and imported into SPSS version 28.00 for descriptive analysis. The mean time spent and percentage of time spent in each of the five domains were calculated. The time spent in the subdomains of diabetes care and non-diabetes were subsequently calculated.

For the qualitative data, interviews were transcribed verbatim and verified for accuracy. Inductive and deductive thematic analysis was conducted in NVivo v12. Two researchers coded the interviews in their original language, and all interviews were anonymised. Open coding was performed, followed by deductive coding, using the conceptual framework constructs that investigators developed based on components from several models found to be pertinent to our study [15, 25]. It also draws on elements of the chronic care model, particularly the organization of healthcare, delivery systems, and leadership support [4]. The framework incorporates both personal and organisational components, which together demonstrate the relationship between personal capacity and systemic support in shaping service delivery. The components of the framework are illustrated in Fig. 1.

Additional codes that were not initially included in the framework but were deemed essential for capturing the full scope of DEs’ tasks were incorporated during analysis. Data saturation was considered reached when no new codes emerged. For publication purposes, the coding was translated into English after the analysis had been completed in the participants’ original language.

## Results

### Quantitative findings

As shown in Table 1, most DEs were nurses with post basic training and had 3–7 years of experience as DEs in PHCs. There were six clinic types, categorized according to daily patient attendance. Type I clinics, which are typically located in population-dense areas, are associated with at least 800 patients per day on average, whereas Type VI clinics, which are usually located in rural areas, are associated with the lowest number of patients, averaging 50 patients per day. The DEs were mostly from Type III clinics, which cater to approximately 300–500 patients per day.

**Table 1:** Participants’ demographic characteristics

Characteristics	*n* (%)
Gender	
• Male	4 (7.8)
• Female	47 (92.2)
Age (years)	Median 35.00 (IQR 5.00)
• 20–30	7 (13.7)
• 31–40	38 (74.5)
• > 40	6 (11.8)
Race	
• Malay	46 (90.2)
• Indian	3 (5.9)
• Chinese	1 (1.9)
• Others	1 (1.9)
Profession	
• Nurse	45 (88.2)
• Assistant medical officer	6 (11.8)
Qualification	
• Advanced Diploma in Diabetes Care	14 (27.5)
• Post Basic Certificate in Diabetes Management	37 (72.5)
Years of service as DE	Median 5.00 (IQR 4.00)
• ≤ 2	10 (19.6)
• 3–7	32 (62.7)
• ≥ 8	9 (17.6)
Clinic type	
• I	2 (3.9)
• II	5 (9.8)
• III	20 (39.2)
• IV	13 (25.5)
• V	9 (17.6)
• VI	2 (3.9)

### Time allocation domain

Over the course of 255 observations, totalling 117,782 min, observations yield five domains of DEs’ activities, namely, (i) Diabetes care, (ii) Non-diabetes care, (iii) Administrative, (iv) Supportive, and (v) Personal. As illustrated in Table 2, a substantial portion of activities were for diabetes care (63.3%), followed by non-diabetes care (26.5%). Administrative, supportive, and personal activities accounted for 4.5%, 3.6% and 2.1%, respectively.

**Table 2:** Distribution of mean time across de’s activities domain

Domain	Sum	Mean time spent, min (SD)	Min-Max	Proportion (%) of total time	95% CI	
					Lower CI	Upper CI
Diabetes Care	74,607	1462.8 (387.6)	522- 2,234	63.3	1353.8	1571.9
Non-diabetes Care	31,180	611.3 (358.5)	25-2077	26.5	510.5	712.2
Supportive	5,261	103.1 (238.7)	0–1,588	4.5	36.0	170.3
Administrative	4,226	82.8 (129.5)	0-597	3.6	46.4	119.3
Personal	2,508	49.1 (94.1)	0-513	2.1	22.7	75.6

**Fig. 2:**
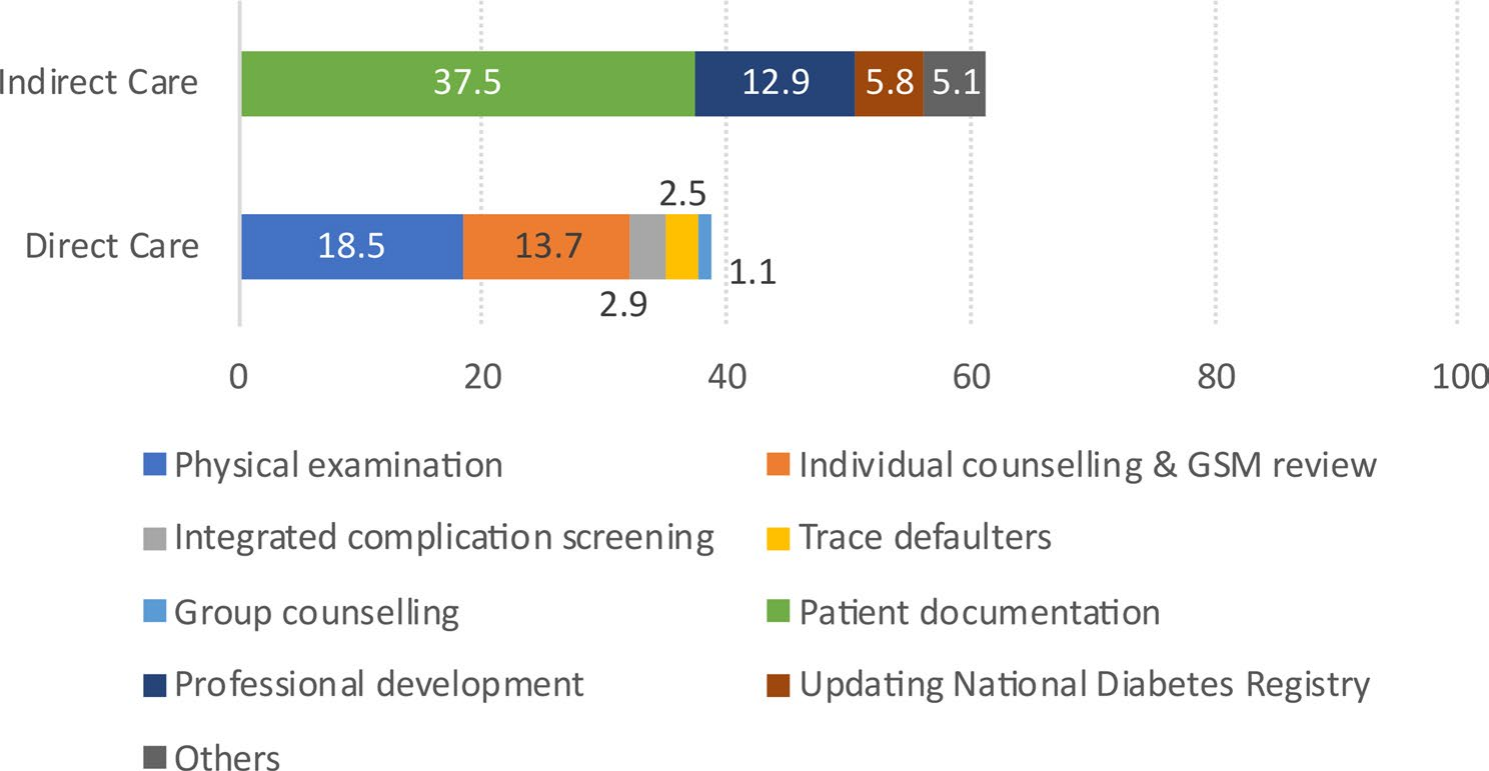
Proportion of time spent for diabetes care activities

**Fig. 3:**
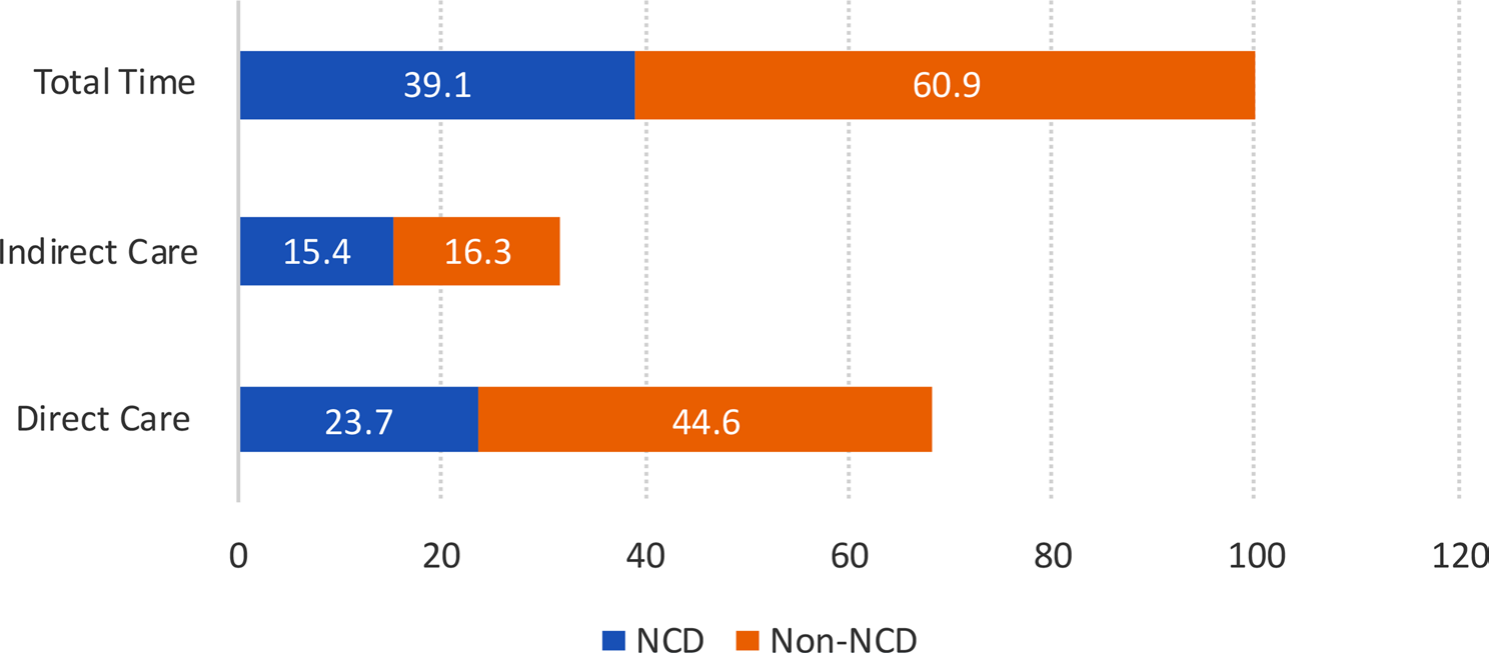
Proportion of time for non-diabetes care activities

For activities involving the diabetes care and non-diabetes care domains—the time proportions were further categorized into direct and indirect patient care, as illustrated in Figs. 2 and 3. The detailed breakdown is provided in Supplementary Table 1.

Figure 2 illustrates the proportion of time DE spent on diabetes care, divided into direct and indirect care. Within the diabetes care domain, more than half (61.3%) of the time was allocated for indirect care, whereas 38.7% of the time was allocated for direct patient care. The most time-consuming indirect activity was patient documentation, which included filling in the patients’ case notes, updating the laboratory results into patients’ files, and charting the patients’ vital signs during the check-up. This is followed by professional development (12.9%) and the updating of the National Diabetes Registry (5.8%). For direct care tasks, activities primarily include physical examination (18.5%), individual counselling and review of self-glucose monitoring (13.7%), and integrated compilation screening and management (2.9%).

In addition to diabetes care, DEs were assigned non-diabetes-related tasks encompassing both NCD and non-NCD tasks, such as mental health screening, triage duties, and emergency room assistance. This accounts for approximately 26.5% of their total working time. The time allocated for non-diabetes care was further dissected and analysed into several subdomains, as illustrated in Fig. 3.

Figure 3 illustrates the proportion of time DE allocated to non-diabetes-related activities, which was further divided into non-communicable disease (NCD) and non-NCD activities. Both direct and indirect care activities are shown. DEs spent a larger proportion of time on non-NCD tasks, particularly within direct care activities. These activities include triage duties, working in emergency room, perform medical examination and administer vaccine. More details on the activities is shown in Supplementary 2.

### Qualitative findings

Thematic analyses identified both facilitators and barriers, with substantial mention of barriers over facilitators. These barriers and facilitators were thematically categorized into personal, organisational, and patient-related factors, each of which influenced their ability to perform their roles effectively. The themes and subthemes are summarized in Table 3. However, despite facing numerous barriers, DEs persevere by leveraging facilitators and utilizing diverse coping strategies.

**Table 3:** Summary of thematic analysis

Theme	Subtheme	Categories	Facilitator (+)/Barrier (-)
Organisational	Workload	Multitasking	(-)
		Staffing resources	(+) & (-)
		Holding multiple portfolios	(-)
		Patient load	(-)
		Documentation amount	(-)
	Leadership support	Understanding DE role	(-)
		Recognition of hard work and value	(+)
		Emotional support	(+)
		Understanding NCD’s importance	(-)
		Supervise and execute responsibility	(-)
		Work delegation and staff allocation	(-)
		Career development and pathway	(+)
	Teamwork	Collaboration with multidisciplinary team	(+)
		Shared understanding of goals	(+)
	Clinic management	Human resource management	(-) & (+)
		Work process and flow	(+)
	Infrastructure	Physical infrastructure	(-) & (+)
		Technical and medical equipment	(-)
	Role conflict	Role conflict	(-)
Patient		Patients’ dependence	(-)
		Patients’ understanding and acceptance	(-) & (+)
		Patients’ appreciation	(+)
Personal	Motivation	Intrinsic needs	(+)
		Religious duty	(+)
		Commitment	(+)
		Acquired skills	(+)

DEs face significant organisational and patient-related barriers that prevent them from delivering optimal care. Organisational challenges emerged as prominent barriers, with workload, leadership support being predominant factors, and role conflict. The next section will present the organisational factors further.

### Barriers in the workload subtheme

A key organisational barrier was the burden of documentation. The DEs highlighted the heavy amount of documentation that patients needed to complete. They highlighted the redundancy of manually recording patient data in both physical books and digital systems. In addition, they spent considerable time on administrative tasks, including the preparation of multiple reports and the completion of patient censuses. This burden consumed valuable time and diverted their attention away from patient-centred activities such as education, counselling, and follow-up care.

*For me*,* it feels overwhelming because you’re expected to use both the book and the TPC (Teleprimary Care) system at the same time. It’s redundant and slows everything down. In a day*,* I’m supposed to be able to see 80 patients*,* but I can only manage 40 or 50 patients because of the time spent writing and typing. It becomes very slow*,* and the service flow is also affected. For example*,* the first patient of the day might only be seen at 9 a.m.*,* even though appointments are scheduled earlier.* (P9, nurse, 8 years DE)

*There are so many things that I have to be in charge*,* not only NCD but also pre-pregnancy (PPC) census*,* where I have to also actively look for patients. So many census. Sometimes*,* I cannot finish the work. Patient attendances in a day*,* sometimes I was not able to fill it in the book. Because I have a lot to do*,* I could not catch up with all these.* (P9, nurse, 8 years DE)

These findings highlight the need for streamlined systems and better digital tools to reduce administrative workloads and enable DEs to focus more on their core roles in diabetes management.

### Barriers in role conflict subthemes

The participants highlighted that the heavy workload in PHCs required them to juggle multiple responsibilities, often beyond their capacity. The DE role compounded this, adding to their existing duties as nurses or AMOs. Managing several tasks simultaneously often hinders their ability to focus fully and fulfil their responsibilities as DE does. One participant, P4, described as follows:

*It was like this: coincidentally*,* that week I was on call*,* and at the same time*,* we had to perform a procedure in the emergency department*,* where as expected*,* there were many patients. Therefore*,* while managing that*,* I also had to rush to the emergency department to assist with the procedure. That’s when things got hectic and overwhelming. Because of that*,* sometimes I couldn’t focus on or carry out my responsibilities as a diabetes educator (DE).* (P4, nurse, 2 years DE)

*During on-call weeks*,* I don’t spend much of my time in the NCD unit. Most of the time*,* I am occupied with procedures and ambulance calls*,* as we handle many ambulance cases here. There are many cases*,* especially at night. Therefore*,* I can only focus on my diabetes duties on Fridays* (P21, medical assistant, 4 years DE).

Role conflict emerged as a significant challenge as participants navigated competing demands between their core responsibilities as DEs and their primary roles as nurses or AMOs. In this context, the expectation of fulfilling both roles created significant barriers for DEs in performing their tasks effectively.

### Barriers in the leadership support subtheme

Additionally, the absence of strong leadership support and limited guidance from supervisors have been repeatedly mentioned as significantly challenging their ability to perform their DE role.

Personal and esteem support: Understanding the role of DE.

The participants emphasized that unclear expectations and a lack of proactive engagement from their supervisors often left them feeling unsupported and uncertain about how to prioritize their responsibilities effectively.

*The supervisor sometimes they just don’t know. This is why they seem uninterested or unaware of the DE’s actual role. For example*,* when I was assigned the two-day schedule*,* the supervisor just said*,* ‘Okay*,* you’ll stay in the NCD unit for those two days to monitor and do the reports.’ Therefore*,* I explained*,* ‘DE’s work isn’t just about doing reports*,* Sister*,* Matron. Let me explain what the job truly involves.’ After I explained it*,* they said*,* ‘Oh*,* I didn’t know. I thought it was just about doing reports.’ Therefore*,* only after we explain things do they understand. Otherwise*,* they just think we’re in the NCD unit doing reports.” Explaining the full scope of DE helps clarify misconceptions*,* but such an opportunity to explain to supervisors is rare* (P18, nurse, 9 years DE).

*To be honest*,* I can count on one hand how many times the supervisor came down to supervise the NCD room… all they cared for was reports. They are more focused on the Pap Smear KPI (key performance indicator). They did not ask about anything related to NCD* (P7, nurse, 5 years DE).

Enabling job support—poor understanding of NCD importance.

The participants also highlighted the poor understanding of the importance of NCDs in the PHC setting. Their enthusiasm for the DE role was often met with resistance from their supervisors, who overlooked the importance of NCD care delivery in PHC compared with other services. While they were likely aware of the increasing numbers of NCD cases, as highlighted in training sessions and projections, concrete actions to support the specific needs of the NCD unit were not always evident.

*If you look at the projections for NCD and mental illness cases*,* NCD cases are skyrocketing. Currently*,* people no longer want to have many children—having one*,* two*,* or three kids is enough. However*,* the number of NCD patients is increasing significantly. However*,* supervisors still do not consider this. Sometimes*,* they are aware; they see the graphs during training sessions showing the rise*,* but there are no concrete steps taken. -* (P1, nurse, 5 years DE)

### Barriers in the infrastructure subtheme

The physical environment further amplified these issues, as the absence of private or suitable spaces for patient education and counselling made it difficult to engage with patients meaningfully. DEs often had to conduct sessions in shared or unsuitable spaces, which could compromise the quality of the interaction and potentially impact patient outcomes.

*The clinic layout lacks privacy; my workspace is in an open area where patients frequently pass through*,* making meaningful counselling sessions difficult.* (P18, nurse, 9 years DE)

The absence of dedicated rooms for DEs and their equipment has also resulted in frequent relocations, which have compromised the functionality and condition of equipment such as fundus cameras.

*For example*,* at Clinic X where I work*,* we used to stay in one room*,* but when the X-ray room was needed*,* we had to move to another room. Then*,* during COVID-19*,* we were moved again to another room. We ended up being relocated so many times that even the fundus camera was damaged. The functioning fundus camera was broken. Moreover*,* to use the fundus camera*,* there needs to be a dedicated dark room. However*,* since we did not have a proper room*,* we had to make do by hanging black cloth around the space to darken it. Eventually*,* it caused the equipment to break.* (P6, nurse, 4 years DE)

In addition to organisational challenges, patient-related factors have emerged as another significant barrier for DEs. These factors, which encompassed patient needs and preferences, understanding and acceptance, and clinic attendance, underscored the multifaceted dynamics of delivering diabetes care. Particularly in rural and underserved settings, the interplay between DEs and patients often introduces additional pressures that further complicate care delivery.

### Barriers in patient factors theme

In rural areas, patients often rely on DEs, expecting home visits even after their mobility improves. This dependency diverted DEs from their clinic duties, creating time constraints and operational challenges that were difficult to resolve despite attempts to transition care back to the clinic setting.

*Living in a rural area*,* patients expect us to visit their homes. When patients expect us to make house calls*,* who will handle our work at the clinic? Therefore*,* we don’t know how to resolve this. Even after discussion with a doctor*,* the doctor also does not know how to provide this service. Like it or not*,* we still have to go to the patient’s house because if we don’t*,* the patients will complain*,* saying we’re not around*,* we’re not being responsible*,* right?* (P4, nurse, 2 years DE)

Additionally, some patients are less receptive to guidance and adhere to their own understanding of diabetes management. This lack of receptiveness may hinder DEs’ efforts to impart meaningful behavioural changes, limiting the effectiveness of their role, building trust, and encouraging collaboration.

*Some patients refuse to follow advice*,* insisting on their own beliefs about managing diabetes*,* which makes behavioural change difficult.* (P7, nurse, 5 years DE)

### Facilitators

Several facilitators within the organisational and patient-related contexts provided significant support to DEs in fulfilling their roles. These included resources and support provided by supervisors as well as from the state health department. While some of these facilitators were workplace specific, such as tailored processes unique to their clinics, others were universally applicable and provided through higher management structures.

### Facilitators in the workload subtheme

One of the key organisational facilitators was adequate staffing and team allocation. Clinics with designated NCD teams or sufficient support staff, such as nurses and AMOs for outpatient departments, enabled DEs to focus exclusively on their roles without being burdened by overlapping duties.

*The reason is the lack of staff*,* as staff nurse S mentioned—there’s a shortage of staff. Therefore*,* while I plan a lot for patients*,* there’s no one to execute it*,* and it ends up incomplete. That’s it. Previously*,* there were two staff nurses*,* including myself. I am the DE*,* and my colleague was the JPL (outpatient department) staff nurse. The JPL staff nurse would handle all the JPL work*,* and we wouldn’t have to address JPL-related tasks. I could focus solely on NCD.* (P13, nurse, 1.5 years DE)

### Facilitators in leadership support

Supportive leadership emerged as another important organisational facilitator. Supervisors who understood the unique responsibilities of DEs and actively acknowledged their contributions fostered a positive and motivating work environment. Emotional support from leaders, such as regular check-ins and assistance with problem solving, helped reduce stress and improve morale.

*The NCD sister truly understands the work we do. Here*,* it is the NCD sister who handles and assigns the annual work target (SKT). They know what tasks we are responsible for and how we are performing. However*,* the sister oversees an entire district*,* so she manages everything on a broader scale. Thus far*,* there haven’t been any issues*,* and the sister has been very supportive.* (P15, nurse, 8 years DE)

Access to training opportunities and professional development was another significant facilitator. Workshops, training sessions, and continuous medical education (CME) programs provide DEs with the skills and knowledge needed to enhance their practice. These platforms also served as opportunities for networking and building a sense of community among DEs, which further contributed to their professional growth and confidence.

*DEs are fortunate to receive consistent training and support from NCD leaders and JKN. Empowerment programs*,* like those in Negeri Sembilan*,* have been instrumental.* (P2, nurse, 7 years DE)

### Facilitators in the teamwork subtheme

Collaboration and teamwork within multidisciplinary healthcare teams were highlighted as essential facilitators. DEs reported that working closely with dietitians, pharmacists, and physiotherapists improved care coordination and patient outcomes. Strong teamwork, supported by shared goals and open communication, created a cohesive work environment that empowered DEs to manage their responsibilities more effectively.

*Therefore*,* there’s also the issue with TCA (To Come Again) appointments*,* where patients often don’t show up. For example*,* if 20 patients are scheduled*,* maybe only 10 will actually come. Therefore*,* what we do is plan with the MO in charge of the NCD clinic. For patients who come in that week*,* we take them right away in the morning for the session on the same day. It’s like a walk-in system*,* where we give them a fast lane. The doctor will see them on the spot during the session*,* check their prescriptions*,* and they can leave earlier. At least they spend approximately one hour attending the session*,* which includes talks by the DE and the pharmacy. Next month*,* we’ll involve physiotherapy and the DE for similar sessions.* (P2, nurse, 7 years DE)

### Facilitators in infrastructure subthemes

Facilities and resources within the workplace, such as dedicated counselling rooms and teaching aids, were critical in enabling DEs to execute their roles efficiently. Having access to private spaces equipped with necessary tools, including fundus cameras and visual aids, improved the quality of patient education and counselling. The participants noted that such resources helped make sessions more engaging and impactful, fostering better patient understanding and adherence to care plans.

*Having a dedicated room equipped with facilities like a fundus camera makes things much easier for all the patients. We can provide consistent instructions to patients*,* and everything is done in one place. This setup also ensures that patients do not wander off or get lost.* (P9, nurse, 8 years DE)

*The room has a TV*,* which makes it much easier for me to give talks than just talking without any visuals. When I use slides*,* the patients can see and follow along more effectively.”* (P21, medical assistant, 4 years DE)

### Facilitators in the clinic management subtheme

The clear processes and structured workflows were identified as key enablers in optimizing DE performance. Standardized procedures, well-defined roles, and effective planning helped streamline tasks, reduce ambiguity, and increase accountability. The participants emphasized that routine workflows and role clarity contributed to smoother coordination of care, minimizing disruptions and improving overall efficiency.

*JKN Negeri Sembilan is highly supportive*,* providing resources such as workflow charts and job descriptions. This clarity helped enhance DE coordination.* (P2, nurse, 7 years DE)

### Facilitators of patient factors

With respect to patient factors, having cooperative and receptive patients facilitated DEs in performing their duties. Patients who were open to communication and willing to follow tailored care plans provided DEs with the flexibility to adapt their approaches to meet individual needs as described by one of the DEs. Even patients who were initially resistant could become accommodating once they listened to the DEs’ advice, and this willingness to cooperate eased the DEs’ workload while contributing to better health outcomes.

*The patient is okay*,* even though they are stubborn. However*,* when we talk to them*,* they listen*,* accept*,* and follow.* (P27, nurse, 9 years DE)

*Even resistant patients eventually follow advice when engaged*,* easing our workload and improving their outcomes.* (P27, nurse, 9 years DE)

Patient appreciation also served as a motivating factor. Instances where patients expressed gratitude for their care deeply inspired DEs to continue their efforts. One DE described how a patient’s weight loss success and gratitude from his wife reinforced their motivation to persist in their role.

*There’s this one patient—He attended counselling sessions with me. I kept following up with him*,* and over time*,* he became motivated. He went from weighing 80 kilograms to being in their 60s. His wife*,* called me to say thank you because her husband has been diligently following my advice. That truly motivated me and gave me the drive to keep going. It was such a boost for my spirit.* (P22, medical assistant, 3 years DE)

### Facilitators in personal factors theme

Despite this external support, DEs understood that they could not rely solely on organisational or patient-related factors to sustain their roles. Instead, they empowered themselves through self-motivation, personal resilience, and individual coping strategies. By taking ownership of their professional growth and adapting to challenges in creative ways, DEs were able to maintain their commitment and manage the complexities of their responsibilities.

DEs demonstrated remarkable resilience and self-motivation in overcoming challenges, relying on various personal factors as key facilitators. Intrinsic motivation emerged as a significant driver, with DEs deriving satisfaction from helping others and improving patients’ health. Some extended this to benefiting their own families, whereas others expressed pride in their work and the positive impact it created.

*The intention is more about helping the community with disease control. We don’t want everyone with diabetes and hypertension to easily suffer from heart attacks or suddenly pass away. With respect to complications*,* over time*,* an increasing number of patients are receiving dialysis. I want to reduce that.* (P21, medical assistant, 4 years DE)

*From the DE course*,* I tried to better understand what patients feel*,* like about insulin and such. We also have family members with diabetes*,* so to some extent*,* we can share input with them. Aside from helping the community*,* we can also help our family members. That’s the purpose.* (P8, nurse, 8 years DE)

DEs also value independence and resourcefulness in fulfilling their responsibilities. They took pride in their ability to analyse patient data, make independent decisions, and take proactive actions without relying on referrals.

*When I screen patients*,* like when the doctor doesn’t refer them*,* I look directly—if their HbA1c was high before or anything. I analyse it myself; anything that doesn’t look okay*,* I address it immediately. I don’t wait for referrals. For example*,* when I see notes such as ‘patient not keen for insulin*,*’ I’ll check directly*,* talk to the patient*,* and promote it right then.* (P17, nurse, 10 years DE)

Commitment to continuous learning further strengthened DEs’ effectiveness. Many have sought opportunities for training, continuous medical education (CME), and self-directed studies to stay updated on diabetes management. A participant with 15 years of experience noted their efforts to attend CME sessions, even outside scheduled invitations, to increase their knowledge.

*I proactively join CMEs when topics are relevant to me*,* even if not invited*,* to stay informed and improve my counselling skills.* (P25, nurse, 15 years DE)

Finally, acquired skills such as building rapport with patients and creating welcoming environments proved to be instrumental. DEs emphasized the importance of psychological approaches such as motivational interviewing and fostering trust to encourage compliance. In addition, P19 described how decorating their counselling room created a calming space for patients, enabling better engagement during sessions.

*Sometimes patients need someone to praise them. As a DE*,* we need to attend courses for counselling. Some also include us in MI (motivational interviewing)*,* so we use some psychology. When we praise patients*,* they feel happy.* (P25, nurse, 15 years DE)

*I’ve probably lasted this long because I enjoy decorating rooms; it’s something I’m interested in. Therefore*,* when patients come in*,* they say*,* ‘This room is so pretty*,* nurse.’ That helps patients feel calm and comfortable talking to me. From there*,* it becomes easier for us to teach and guide them.* (P19, nurse, 11 years DE)

These personal facilitators, such as motivation, appreciation, religious conviction, independence, commitment to learning, and acquired skills, have enabled DEs to remain resilient and focused. They not only overcame barriers but also continued to deliver impactful care, demonstrating their adaptability and dedication in challenging circumstances.

These organisational facilitators highlight the importance of a supportive environment in enabling DEs to perform their roles in diabetes care and contribute to managing the growing burden of noncommunicable diseases.

## Discussion

This study provides one of the first mixed-methods examinations of the roles and time utilisation of DEs in Malaysian PHCs. By integrating time-motion observations with qualitative findings, the study offers a comprehensive understanding not only of how DEs allocate their working time, but also how organisational structures, workforce arrangements, and service delivery priorities shape their role in practice. In general, the findings indicated that while DEs are more engaged in diabetes care, how this engagement is delivered and its outcomes are influenced by existing organisational structure and workflows.

The quantitative findings showed that diabetes-related activities accounted for the majority of DEs’ working time. However, a large proportion of this time was spent on indirect care, particularly documentation-related tasks. This pattern suggests that although diabetes care remains a central component of DEs’ responsibilities, direct patient-facing activities such as counselling and education are constrained by administrative demands. Furthermore, qualitative findings provided important contextual explanations for this observation. DEs described extensive documentation demands involving multiple reporting platforms, including paper-based records and electronic systems. Similar challenges have been reported in primary care settings internationally, where this documentation burden not only limits DEs’ productivity but also contributes to inefficiencies within the healthcare system [26, 27]. Ideally, patient care should remain the primary focus of DEs’ work.; however, in countries such as the United Kingdom and Australia, DE usually operate within more integrated information systems and clearer reporting structures, allowing a greater proportion of their time to be allocated to direct patient education and follow-up [28, 29]. In contrast, the Malaysian context involves more extensive documentation processes within current administrative arrangements. To address this, future interventions and healthcare policies should aim to optimize documentation processes. Potential strategies include implementing streamlined electronic health record systems across all clinics and referral centres [30, 31], introducing standardized templates, and providing clear guidelines on work processes [32].

In addition to diabetes-related work, DEs spent a considerable proportion of their time on non-diabetes care activities. Qualitative data indicated that this was largely attributable to role conflict arising from DEs’ concurrent responsibilities as nurses or assistant medical officers. The expectation to fulfil multiple clinical roles within resource-constrained PHCs resulted in fragmented work patterns and reduced capacity to prioritise diabetes education. This situation is further aggravated by poor understanding among supervisors and other HCWs of the importance of DE in diabetes management [15, 33]. This contrasts with workforce arrangements in countries such as Australia and the UK, where DE are typically appointed to clearly defined specialist roles, with minimal overlap with general nursing duties [28, 29, 34]. This dual-role arrangement aligns with current operational norms in Malaysian primary healthcare, where staff are often required to support multiple service areas. From a service delivery perspective, this barrier may reduce continuity of care and depth of diabetes education, potentially affecting long-term disease management outcomes. Hence, implementing protected role boundaries in these settings reduce task dilution and support continuity of diabetes care [35, 36], and additional efforts should be made to improve HCWs’ understanding of the role of DEs and their significant contribution to diabetes management in PHCs.

Leadership support emerged as a key organisational factor influencing DEs’ ability to perform their roles effectively. Participants described insufficient supervision and limited understanding of the DE role among clinic leaders, with some mentioning that diabetes and other NCD services received limited recognition within primary care. This lack of leadership engagement affected task allocation, supervision practices, and access to resources. In contrast, clinics with supportive leadership, clearer delegation, and dedicated NCD teams appeared better able to facilitate DEs’ work. Similar observations have been made in health systems such as the United Kingdom and Australia, where diabetes education is explicitly recognised as a core component of chronic disease management and supported by defined performance indicators and professional governance frameworks [5, 29, 37].

The lack of effective supervision also emerged as a key barrier. Participants highlighted the absence of regular feedback, limited managerial understanding of NCD care, and inadequate support mechanism. This finding was also seen in another study showing how inadequate leadership attention acted as an important barrier and undermined their morale as well as performance [32, 38, 39]. These findings highlight the importance of leadership awareness and engagement in shaping service delivery at the primary care level [39]. Enhancing leaders understanding of NCD care and recognising the contribution of DEs could serve as a key strategy to improve workforce efficiency and service quality [4, 40, 41].

Despite these organisational challenges, DEs demonstrated notable resilience, driven by intrinsic motivation, professional commitment, and positive patient feedback [22]. This resilience enabled them to continue delivering diabetes education under demanding conditions. Similar traits have been observed among DEs internationally, though clearer roles and stronger support systems elsewhere help sustain their resilience [35, 42]. From a health services perspective, an over-reliance on individual resilience in the absence of adequate organisational support raises concerns regarding long-term workforce sustainability [42, 43]. In Malaysia, multiple studies have shown that high workloads, stressful working conditions, and limited organisational support are associated with increased burnout and workforce strain among healthcare workers [43, 44]. In addition, recent local evidence indicates that role ambiguity is a significant predictor of burnout within Malaysia’s public healthcare sector [45]. Evidence from international integrated diabetes care services suggests that organisational support plays an important role in sustaining specialist diabetes roles and service delivery [35].

Collectively, the findings highlight a need to better align the intended role of DE with operational contexts in Malaysian PHCs. Key areas for improvement include reducing documentation burden through more integrated information systems [26, 30, 46], clarifying DE roles within clinic staffing models and strengthening team-based chronic disease care [15, 40, 47], enhancing leadership engagement to support NCD service delivery [22, 48], and supporting structured professional development pathways for HCPs involved in diabetes care [49]. Addressing these areas may improve service efficiency and the consistency of diabetes education, and strengthen primary healthcare responses to Malaysia’s growing diabetes and NCD burden [17, 20, 24].

## Conclusion

This study is among the first in Malaysia to quantify how DEs allocate their time and explore the factors shaping their role in primary healthcare. The findings of this study offer a baseline for strengthening the DE workforce in Malaysia and similar LMIC settings. In LMIC such as Malaysia, primary healthcare often faced resource constrains, high patient volume and underdeveloped technological support. Thus, while DEs dedicate most of their time to diabetes care, much of it is consumed by indirect activities such as documentation and competing nursing responsibilities. Organisational barriers such as high workload, limited leadership support, and role conflict, further constrain their effectiveness, although facilitators such as teamwork, training opportunities, and patient appreciation help sustain their efforts. Using a mixed-methods approach, the findings show that while DEs remain substantially engaged in diabetes care, the delivery and impact of their work are influenced by documentation burden, role overlap, and leadership support within primary care settings. These findings highlight priorities and practical ways to strengthen diabetes education services. These include reducing documentation workload by integrating information systems, providing clearer role definitions with dedicated diabetes-focused time, increasing leadership engagement with noncommunicable disease services, and providing structured opportunities for professional development. Supporting DEs through organisational and managerial support, rather than relying on individual effort alone may improve service efficiency, strengthen workforce sustainability, and enhance primary healthcare responses to the growing diabetes burden in Malaysia and similar LMIC contexts. Future research should assess the effectiveness of such interventions in improving diabetes education services and continuity of care.

## Limitations

There were several limitations to this study. The observations conducted by the researchers include only the five working days for the week. Any activities performed during weekends and public holidays, especially within the surrounding community, were not recorded. The conduct of DEs may be altered by the presence of observers, which is known as the Hawthorne effect. As previous studies have shown that the effect attenuated over a short period of time, given the time and length of observation per DE in this study, the researchers have no reason to believe that the behaviour of the DEs was meaningfully altered throughout the course of the data collection. Consequently, researchers have taken all possible measures to minimize this effect.

In addition, although DEs were recruited from clinics of different types and sizes, the analysis was not stratified by clinic type. As a result, potential differences in time utilisation related to clinic size, patient volume, and service scope could not be examined. Finally, this study was conducted in a single state, and its findings may limit generalizability to other states in Malaysia. Nevertheless, the results provide valuable insights into the roles and challenges of DEs in public primary healthcare and may inform future research and policy considerations in similar contexts. Regardless, its results can still be informative and provide valuable insights to researchers and practitioners in different locations.

## Supplementary Information

Below is the link to the electronic supplementary material.

Supplementary Material 1

Supplementary Material 2

Supplementary Material 3

## Data Availability

All relevant data are included in the manuscript. Additional data generated and analysed during the current study are available from the corresponding author upon reasonable request.

## Abbreviations

AMO: Assistant Medical Officer
BP: Blood Pressure
BMI: Body Mass Index
C&P: Credential & Privileging
CME: Continuous Medical Education
DM: Diabetes Mellitus
DE: Diabetes educator
DSME: Diabetes Self-Management Education
ECG: Electrocardiogram
ECHO: Echocardiogram
HCP: Healthcare Provider
HPT: Hypertension
MCH: Maternal and Child Health
MC: Medical Certificate
MO: Medical Officer
MOH: Ministry of Health
NCD: Noncommunicable Disease
NDR: National Diabetes Registry
OPD: Outpatient Department
PHC: Primary Health Clinics
