# Supplementary material for: Quantifying and understanding the roles of diabetes educators in Malaysian primary health clinics: a mixed-methods study
Source: BMC Health Serv Res. 2026 Feb 3;26:236. doi: 10.1186/s12913-026-14144-7 (PMC12903666; doi:10.1186/s12913-026-14144-7)
Supplement: Supplementary file 1 — Supplementary Material 1 [file 12913_2026_14144_MOESM1_ESM.docx]

Appendix A: Interview Guide

| **Domain** | **Sub-domain** | **Question** |
| --- | --- | --- |
| Personal | Knowledge | How do you feel about the knowledge that you gained throughout the six months of diabetes management course?  Probe:   - Do you feel it is adequate to prepare you to deal with patients in the clinic? - Is there any other additional training or course that you attended to enhance your knowledge of diabetes? - Do you have any issues & challenges with performing DE tasks with your current knowledge? |
|  | Commitment | How do you manage tasks between diabetes care tasks and other tasks?  Probe:   - How do you divide your time? - How do you prioritise the tasks? - What motivates you to perform diabetes tasks even though you are also required to do other tasks? |
|  | Multitasking | What do you think of your workload as a DE?  Probe:   - How do you divide time between diabetes and non-diabetes tasks? - Given constrained time, what aspect of diabetes care that you feel is the most essential to do? - Do you have any issues & challenges with performing DE tasks with your current skills? |
|  | Acquired Skills | Do you feel you have the skills needed to provide patients with diabetes education and care? Probe:   - What skills do you think are important as a DE? - Do you have any issues & challenges with performing DE tasks with your current skills? |
| Organisation | Workplace Workload | What do you think about your current workload as a nurse / Assistant Medical officer (AMO)?  Do you think that the current workload influences your time to perform DE task?  What do you think of your workload as a DE? |
|  | Leadership support | How do you find the support from your management and supervisors?  Probe:  Supervisors - *Nursing supervisor/Head of nursing supervisor/medical officer in-charge/Family Medicine Specialist   - Who decides on your duties, your roster? - Do the supervisors know you are DE? - Do they consider your role as DE in any project or team? - Do they guide you during performing your task as nurse /AMO and / or as DE |
|  | Local teamwork | Do you have a dedicated team for diabetes in the clinic?   - If yes, what are the duties of each team member?   How about the support from other staff? E.g administration/attendants/other nurses/AMO?  Do they also help you in some part of your duties e.g registration, vitals sign or filing? |
|  | Supportive Infrastructure | Do you have the conductive infrastructure and facilities for diabetes care and consultation?  Probe:   - Do you have any issues & challenges with performing DE tasks with your current workplace? - What facilities do you hope for a better service? |
|  | Policies and Practices | Is there any local policy or ruling that affects your duties as a DE?  Probe:   - Who sets up the policy? |
|  | Strategic Priorities? | Is there any other program in the clinic that you are involved in?  Probe:   - Describe in brief what are the programs and what are your roles in it? - How has that affected your time for diabetes care? - Do you have any issues & challenges with performing DE tasks with your current project priorities? |
