# Supplementary material for: Quantifying and understanding the roles of diabetes educators in Malaysian primary health clinics: a mixed-methods study
Source: BMC Health Serv Res. 2026 Feb 3;26:236. doi: 10.1186/s12913-026-14144-7 (PMC12903666; doi:10.1186/s12913-026-14144-7)
Supplement: Supplementary file 2 — Supplementary Material 2 [file 12913_2026_14144_MOESM2_ESM.docx]

Supplementary File 1:

Table 1: Activities Within Diabetes Care Domain

|  | **Subdomains of diabetes care** | **Mean time spent, min (SD)** | **Proportion (%)** | **Total %** |  | | |
| --- | --- | --- | --- | --- | --- | --- | --- |
| Direct Care | Physical examination | 269.9 (163.1) | 18.5 | 38.7 | |  |  |
|  | Individual counselling and glucose self-monitoring review | 196.5(196.2) | 13.7 |  |  |  |  |
|  | Integrated complication screening and management | 41.3 (54.2) | 2.9 |  |  |  |  |
|  | Trace defaulters | 36.4 (56.2) | 2.5 |  |  |  |  |
|  | Group counselling | 15.4 (42.7) | 1.1 |  |  |  |  |
| Indirect Care | Patient documentation | 547.8 (266.6) | 37.5 | 61.3 | |  |  |
|  | Professional development | 188.2 (343.3) | 12.9 |  |  |  |  |
|  | Updating National Diabetes Registry (NDR) | 83.5 (164.9) | 5.8 |  |  |  |  |
|  | Others | 74.9 (109.6) | 5.1 |  |  |  |  |
| TOTAL | | | | 100 | |  |  |
