# Supplementary material for: Quantifying and understanding the roles of diabetes educators in Malaysian primary health clinics: a mixed-methods study
Source: BMC Health Serv Res. 2026 Feb 3;26:236. doi: 10.1186/s12913-026-14144-7 (PMC12903666; doi:10.1186/s12913-026-14144-7)
Supplement: Supplementary file 3 — Supplementary Material 3 [file 12913_2026_14144_MOESM3_ESM.docx]

Supplementary File 2

Table 1: Activities Within Non-Diabetes Care Domain

| \| Subdomains of non-diabetes care \| \| Mean time spent (SD) \| Proportion (%) \| \| --- \| --- \| --- \| --- \| \| Direct Care NCD \| Anthropometry measurement for other NCD patients e.g. hypertensive \| 96.9 (135.7) \| 23.7 \| \| Vital signs measurement \| \| Dietary and medical advice \| \| Direct Care Non-NCD \| Mental health screening \| 182.4 (9304.0 \| 44.6 \| \| Triage duties \| \| On duty at emergency room \| \| Administer vaccine \| \| Perform medical check-up \| \| Perform visual acuity test \| \| Assess color-blindness \| \| Indirect Care NCD \| Open hypertension patient’s card \| 62.7 (3200.0) \| 15.4 \| \| Update monthly census on NCD \| \| Update patient’s medication and laboratory results in patient’s card \| \| Indirect Care Non-NCD \| Register patient at the clinic counter \| 66.8 (3405.0) \| 16.3 \| \| Arranging patients’ laboratory results \| \| Label and color coordinate patients’ card \| \| Distribute patients’ card to doctor \| \| TOTAL \| \|  \| 100 \| |
| --- | --- | --- | --- | --- | --- | --- | --- | --- | --- | --- | --- | --- | --- | --- | --- | --- | --- | --- | --- | --- | --- | --- | --- | --- | --- | --- | --- | --- | --- | --- | --- | --- | --- | --- | --- | --- | --- |
